# Anti-SARS-CoV-2 mRNA vaccination among patients living with SLE in Sweden: Coverage and clinical effectiveness

**DOI:** 10.1177/09612033241273052

**Published:** 2024-08-12

**Authors:** Arthur Mageau, Julia F Simard, Elisabet Svenungsson, Elizabeth V Arkema

**Affiliations:** 1 Department of Medicine Solna, Clinical Epidemiology Division, 271345 Karolinska Institutet, Stockholm, Sweden; 2 Département de Médecine Interne, Assistance Publique Hôpitaux de Paris, INSERM IAME UMR 1137 Team Descid, Université Paris Cité, Paris, France; 3 Department of Epidemiology & Population Health, 6429 Stanford University School of Medicine, Stanford, CA, USA; 4 Division of Immunology and Rheumatology, Department of Medicine, 6429 Stanford University School of Medicine, Stanford, CA, USA; 5 Department of Medicine Solna, Division of Rheumatology, 27106 Karolinska Institutet, Karolinska University Hospital, Stockholm, Sweden

**Keywords:** Vaccination, SARS-CoV-2, vaccination effectiveness

## Abstract

**Objectives:**

To describe the uptake of anti-SARS-CoV2 vaccination in 2021 and investigate vaccine effectiveness in systemic lupus erythematosus (SLE) patients in Sweden.

**Methods:**

The cumulative incidence of first anti-SARS-CoV2 vaccination was estimated among SLE patients from the Swedish National Patient Register and matched comparators living in Sweden on January 1, 2021. To assess vaccine effectiveness, we included the individuals who received two doses of anti-SARS-CoV2 mRNA vaccines before year 2022, with no COVID-19 diagnosis code before the 2nd vaccine dose. Hospitalization rates with COVID-19 as main diagnosis during the year after second dose were compared between SLE patients and comparators in multivariable-adjusted marginal Cox models, overall and stratified by immunosuppressive treatment received during the year before second vaccine dose.

**Results:**

Vaccination uptake was similar between SLE patients and comparators. By December 2021, 9% of both SLE and comparators had not received any vaccine doses. Among 5585 SLE patients and 37,102 comparators, 11 COVID-19 hospitalizations in the SLE group and 20 in the comparators occurred. SLE was associated with a higher risk of COVID-19 hospitalization (HR = 3.47, 95%CI 1.63-7.39). The HR was higher for immunosuppressive-treated SLE (7.03 95%CI 3.00-16.46) than for immunosuppressive-untreated (1.50 95%CI 0.34-6.60). Vaccination of immunosuppressive-untreated SLE patients had similar effectiveness as comparators.

**Conclusion:**

Anti-SARS-CoV2 vaccination coverage was similar between SLE patients and the general population in Sweden. Even though the incidence of post-vaccination COVID-19 hospitalization was very low, vaccine effectiveness was diminished in SLE patients compared to the general population and lowest in those treated with immunosuppressants.

## Key points

### What is already known on that topic

Several reports showed that the humoral response following vaccination was lower in SLE patients than the general population. However, real-world data assessing the effectiveness of the vaccination using clinical outcomes in this population are currently lacking.

### What this study adds

We leveraged the power of Swedish national registers to describe vaccine uptake and examine its clinical effectiveness in the SLE population living in Sweden. Among over 5000 SLE patients and 37,000 matched comparators who received two doses, we observed SLE was associated with a 3.5-fold higher risk of COVID-19 hospitalization (hazard ratio 3.47, 95%CI 1.63-7.39). Vaccination of immunosuppressive-untreated SLE patients had similar effectiveness as comparators.

### How this study might affect research, practice or policy

The vaccination scheme could possibly be tailored among patients treated with immunosuppressants. The use of other prophylactic treatment such as monoclonal antibodies should be proposed to immunosuppressant-treated SLE patients in case of reactivation of the pandemic or if mRNA vaccines are developed for other indications.

### Patient and Public Involvement statement

Patient or public were not involved in the design or the conduction of the study.

### Competing interests

The authors declare no potential conflict of interest with this work.

## Introduction

The COVID-19 pandemic has raised particular concerns for patients with systemic lupus erythematosus (SLE) who are often immunocompromised, more prone to infection and may have several comorbidities such as chronic kidney disease.^1,2^ Furthermore, SLE has been shown to be associated with a poor prognosis after a COVID-19-associated organ failure, independently of major comorbidities.^3^ Therefore, anti-SARS-CoV-2 vaccination was quickly recommended in the SLE population.^4^ Unfortunately, several immunological reports showed that the production of a humoral response following vaccination was lower in SLE patients than the general population, even for patients without immunosuppressive treatments.^5,6^ However, the humoral response measured by the concentration of vaccine-induced anti-spike (anti-S) antibodies, which has been the most studied, is only a surrogate marker of vaccine effectiveness. Thus, some studies showed that immunocompromised patients could have a T-cell-driven vaccine response even without a humoral response.^7^ Unfortunately, real-world data assessing the effectiveness^8^ of the vaccination using clinical outcomes in this population are currently lacking. Furthermore, the impact of immunosuppressive treatments has not been studied at a large scale and with long-term follow-up.

Our overall aim is to determine whether, to which extent and among which patients, anti-SARS-CoV2 mRNA vaccination clinical effectiveness is affected by SLE. Specifically, we aim to answer the following questions: (i) Was the uptake of the SARS-CoV2 mRNA vaccination similar between SLE patients and general population? (ii) Do vaccinated SLE patients have an increased risk of hospitalization for COVID-19 compared to vaccinated people without SLE from the general population? (iii) Among patients with SLE, does the risk of COVID-19 hospitalization vary by immunosuppressive treatment? A better understanding of the vaccination effectiveness and of the factors associated with a decreased effectiveness could help to improve the vaccination guidelines in this population.

## Methods

### Study settings and data source

In Sweden, the healthcare system is tax-funded and universally accessible to residents. Data generated by interaction with the healthcare system are captured in registers and can be linked using an individual’s unique identification number. We created a large cohort of individuals with and without SLE by linking several nationwide and population-based registers. 10 randomly sampled comparators from the general population without SLE were identified in the Total Population Register, and matched with SLE cases on age, sex, calendar time, and county of residence. We collected information on hospitalizations and outpatient visits to specialist care from the National Patient Register (NPR; nationwide coverage of hospitalizations since 1987 and of outpatient visits since 2001). Data quality is high,^9^ but results of biological, histological or imaging examinations are not available in the NPR. Prescription medication dispensations were obtained from the Prescribed Drug Register (PDR) - which was available starting July 2005 for the entire population - and from the Swedish Rheumatology Quality (SRQ) register,^10^ which covers a part of the SLE population in Sweden since 2007. Anti-SARS-CoV2 vaccination data were obtained from the National Vaccine Register. Offspring data (number of children, date of their birth) were obtained from the Multigeneration Register (MGR). Follow-up in all of the registers was through Dec 31, 2022.

### Study populations

Using inpatient and outpatient visit data in the NPR, SLE was defined according to the previously-established Definition^11^ as ≥ 2 ICD-coded visits with ≥1 code from a specialist who typically treats or diagnoses SLE (i.e., rheumatology, dermatology, nephrology, internal medicine, or pediatrics). General population comparators had no SLE codes before the date of first observed SLE ICD-coded visit of their matched case.

We studied two different populations: one to describe uptake of the vaccination (Population 1) and another one to assess vaccine effectiveness (Population 2).

In population 1, we included all individuals ≥18 years old living in Sweden at the start of the vaccination campaign (January 1st, 2021) who fulfilled the definition of SLE before January 2021 and their matched comparators.

In population 2, we included all individuals who fulfilled the definition of SLE before January 2021, who were vaccinated according to the standard scheme. Individuals (SLE and matched comparators) were selected if they received two doses of anti-SARS-CoV2 mRNA vaccines before January 1^st^, 2022, if they were ≥18 years old at first vaccine dose, if there were more than 10 days and less than 90 days between the two injections and if they did not receive any COVID-19 diagnosis code in the NPR before the 2nd vaccine dose.

### Outcomes

For the description of vaccination uptake, the main outcome was first vaccine injection.

For the vaccine effectiveness assessment, the main outcome was a first hospitalization with COVID-19 (defined as ICD-10 codes U07.1 or U07.2) as main diagnosis for the hospitalization listed in the inpatient component of the NPR. The secondary outcome was defined as first COVID-19 diagnosis in inpatient or outpatient care, as main or secondary diagnosis.

### Follow-up

For the description of the vaccination coverage, the follow-up period was the year 2021 (January 1st -December 31st).

For the vaccine effectiveness assessment, start of follow-up was defined as the date of the second dose of the anti-SARS-CoV-2 mRNA vaccine. End of follow-up was the date of the first in-hospital admission for COVID-19, death, emigration, third vaccine dose or 12 months after start of follow-up, whichever came first. We censored individuals at third vaccine dose because our main interest was the clinical effectiveness of the first two doses and because the time between the second and the third dose varied considerably between patients. We defined a global censoring at 12 months after second dose if none of the listed events occurred because we assumed that the effect of the first two doses was not supposed to last longer.

### Covariates

Among all the included individuals, we defined subpopulations according to the disease-modifying treatments that they received and according to their comorbidities. We defined the use of an oral treatment if a patient had two dispensations in the Prescribed Drug Register of the same drug during the year before start of follow-up (i.e. January 1st 2021 for vaccination coverage description and date of second dose for vaccine effectiveness assessment). For treatment given by infusion (belimumab, rituximab and cyclophosphamide) we defined the use if a patient had one infusion during the last 6 months before inclusion. Data on infusions were obtained from both the NPR (infusion procedure code associated with a ATC code of interest) and SRQ. Of note, these registers do not have complete coverage of infusions as some of them might not be reported in the NPR and SRQ does not have 100% coverage of SLE patients in Sweden. Out-of-hospital dispensations of belimumab were also retrieved from the PDR, and considered as infusions.

Comorbidities (chronic kidney disease, obesity, and arterial hypertension) were defined as any history of relevant ICD codes and at any time before inclusion. Diabetes was defined as any ICD code or any use of an antidiabetic drug before inclusion.

Civil status from the Total Population Register and age of offspring from the Multigeneration Register were used to derive a three-level variable for household composition: (i) living alone, (ii) living with a partner, without any child <18 years old, and (iii) having child (ren) < 18 years old, with or without partner. See electronic Supplemental Materials (ESM), Table S1.

### Statistical analyses

Characteristics of the study populations were compared by exposure (SLE vs general population) using frequencies and median with first (q1) and third quartile (q3).

To describe the vaccination campaign course, we calculated and plotted a cumulative incidence function using first vaccine injection as outcome of interest and death or emigration as censoring events. We described the characteristics of the SLE population remaining unvaccinated on December 31^st^, 2021, with descriptive statistics and compared them to those SLE patients who get vaccinated.

We studied the vaccine effectiveness with a time-to-event analysis using a marginal Cox proportional hazard model that considers the matching between SLE and matched comparators.^12^ The Cox model estimated the hazard ratio (HR) and 95% confidence intervals (95%CI) of COVID-19 associated with SLE diagnosis compared to non-SLE comparators. The model was adjusted for age, sex, health administrative region and household composition. We used household composition as a covariate in the multivariable analysis because we considered this variable strongly associated with the risk of being in contact with SARS-CoV-2. We did not adjust for SLE-related comorbidities such as chronic kidney disease because we considered them as part of the SLE global burden.

To study the risk according to the prior treatment received, we stratified SLE patients by use of immunosuppressive treatment in the year before the second vaccine dose was received. Hydroxychloroquine was not considered an immunosuppressive drug. We plotted the survival without COVID-19-related hospitalization in SLE patients with and without immunosuppressive treatment as well as in general population without immunosuppressive treatment according to the Kaplan-Meier method. Then, we ran a standard Cox regression model to calculate the HR of COVID-19 hospitalization associated with SLE with or without immunosuppressive treatment, using general population without immunosuppressive treatment use at start of follow-up as reference. Here, we included age, sex, health administrative region, household composition and number of contacts with the healthcare system in 2020 as covariates. Finally, we described more precisely which immunosuppressive drugs were used in the year before vaccination by patients that experienced the main outcome with descriptive statistics.

### Sensitivity analyses

We ran several sensitivity analyses. First, to examine whether informative censoring affected our results, we used inverse probability censoring weighting (IPCW).^13^ We modelled the probability of being censored because of a third vaccine dose using age, place of birth (Sweden or another country), household composition, type of vaccine (Comirnaty® vs Spikevax®) and number of contacts with the healthcare system in 2020 as baseline covariates. Calendar period (before or after January 1^st^, 2022) was used as a time-dependent covariate. Weights obtained using this modelling were used in the marginal Cox model. Second, we did not censor patients at third vaccine dose and kept following them until date of the first in-hospital admission for COVID-19, death, emigration, or 12 months after start of follow-up, whichever came first.

In another sensitivity analysis, we added a time-dependent covariate in the marginal Cox model to take into account the COVID-19 wave in Sweden between November 18^th^, 2021 and April 20^th^, 2022.^14^ We looked for an interaction between SLE and the wave period.

The study protocol was registered to OSF registries https://doi.org/10.17605/OSF.IO/RSCBZ

Ethical approval was provided by the Regional Ethics Review Board in Stockholm (DNR 2021-01,148). Informed consent was not required.

## Results

### Vaccine coverage progression (population 1)

**Figure 1.**
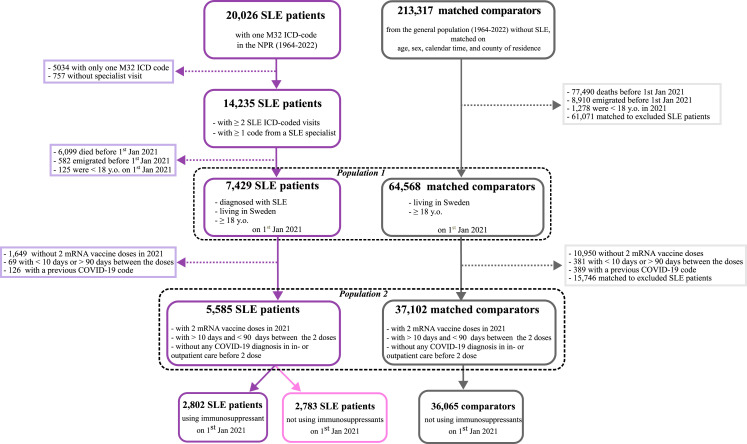
Flow-chart of the study NPR: National Patient Register; SLE: Systemic lupus erythematosus; Jan: January; y. o.: years old; ICD: International Classification of Diseases.

We identified 7429 adults living in Sweden and diagnosed with SLE according to our definition on January 1^st^, 2021. These patients were matched to 64,568 comparators living in Sweden and ≥18 years old on the same date. A more detailed flow chart of the study is presented in Figure 1. The median [q1-q3] age was 57.0 [44.0-70.5] years at start of follow-up in the SLE group and 55.9 [43.1-68.9] among comparators. 86.7% were female in the SLE group and 87.1% in the non-SLE group.

**Figure 2.**
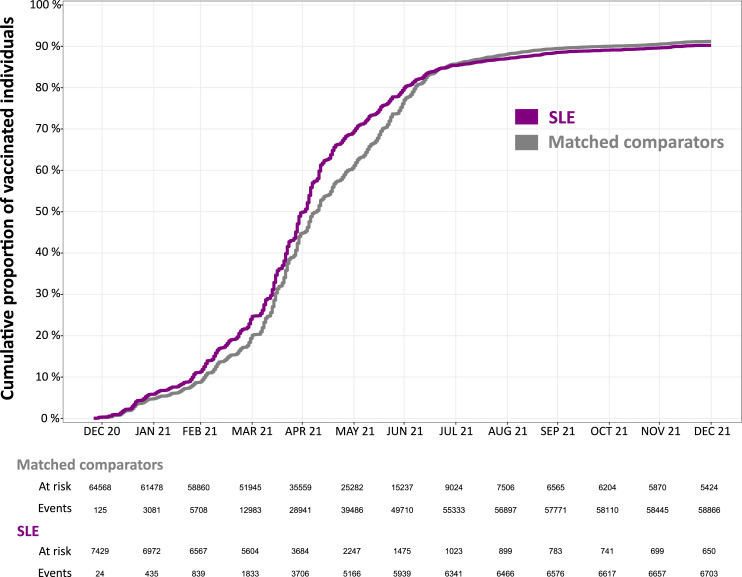
Progression of the vaccine coverage in SLE patients compared to matched comparators in Sweden December 2020 to December 2021. SLE: systemic lupus erythematosus.

The cumulative incidence of anti-SARS-CoV-2 vaccination in the SLE and comparator groups is displayed in Figure 2. Few doses (24 in the SLE group and 125 in the comparator group) were administered as early as December 2020, and were included in the analysis, but most of the first doses were administered in spring 2021. We observed similar trends in vaccine uptake in SLE and comparators. The maximum difference between the two groups was observed at the end of May 2021 when 70% of the SLE patients and 60% of the comparators had received a first dose. After the end of September 2021 very few first doses were newly administered and the slope of the cumulative incidence function became almost flat for both groups.

On December 31^st^, 2021, 650 SLE patients remained unvaccinated. The characteristics of these patients compared to the vaccinated SLE patients at that time are presented in ESM, Table S2. Briefly, unvaccinated SLE patients were younger (median [q1-q3]: 47.4 [35.6-58.5] years compared with 57.9 [45.0-71.0] years in the vaccinated group) and more frequently born outside of Sweden (34.8% in the unvaccinated group vs 16.4% in the vaccinated group). One possible reason for not being vaccinated at that time was having a previous COVID-19 infection. We observed that 6.0% of the unvaccinated and 3.0% of the vaccinated had an inpatient or outpatient visit listing a COVID-19 diagnosis code during 2021. It should be noted that we do not have information on positive tests in this study, nor visits in primary care, and are likely underestimating previous COVID-19 infection.

### Vaccine effectiveness (population 2)

For the assessment of vaccine effectiveness, we included 5585 SLE patients and 37,102 matched comparators from population one who also met the following criteria: having received two mRNA vaccine doses before January 1^st^, 2022, with >10 days and <90 days between the two doses and without any COVID-19 diagnosis in the NPR before second dose (Table 1). Both groups received mostly the Comirnaty® vaccine (87.4% in the SLE group and 87.2% in the comparator group). As expected, the individuals with SLE had more contact with the healthcare system in 2020 and more comorbidities, such as chronic kidney disease (5.8% in the SLE group vs 0.6% in the comparator group. Half (50.2%) of the SLE patients were using an immunosuppressive treatment before their 2^nd^ vaccine dose, the majority of whom had received corticosteroids (75.8%). Only 2466 (44.2%) SLE patients had been dispensed hydroxychloroquine twice during the year before second vaccine dose.

**Table 1.** Baseline characteristics of the SLE patients and matched non-SLE comparators from the general population in Sweden who received two doses of mRNA vaccines in Sweden in 2021, and before any COVID-19 diagnosis in in- or outpatient care (population 2).

	SLE *N* = 5 585	NON-SLE *N* = 37 102
Demographics		
Age on Jan 1^st^, 2021, years, median [q1-q3]	57.0 [44.7-70.4]	55.2 [44.0-66.7]
Female, *n* (%)	4 819 (86.3)	32 113 (86.5)
Born out of Sweden, *n* (%)	914 (16.4)	4 772 (12.9)
Education level, *n* (%)		
0-9 years	980 (17.5)	4 978 (13.4)
10-12 years	2 358 (42.2)	15 952 (43.0)
≥ 10-13 years	2 213 (39.6)	16 004 (43.1)
missing	34 (0.6)	168 (0.5)
Household composition, *n* (%)		
Living alone	2 412 (43.2)	14 715 (39.7)
Having child(ren) < 18 years old	1 246 (22.3)	10 587 (28.5)
Living with a partner, without having any child < 18 years old	1 927 (34.5)	11 800 (31.8)
Health administrative region, *n* (%)		
Stockholm	1 137 (20.4)	7 145 (19.3)
Uppsala-Örebro	1 215 (21.8)	8 187 (22.1)
South	913 (16.3)	5 840 (15.7)
West	1 038 (18.6)	7 047 (19.0)
North	551 (9.9)	3 847 (10.4)
Southeast	731 (13.1)	5 036 (13.6)
Number of contacts in inpatient or non-primary outpatient care in 2020, median [q1-q3]	3 [1-7]	0 [0-2]
Vaccine		
Comirnaty® (pfizer)	4 879 (87.4)	32 335 (87.2)
Time between the two doses, in days	42 [34-43]	42 [40-47]
SLE		
Time since SLE diagnosis, in years, median [q1-q3]	13.0 [6.6-19.2]	
Comorbidities		
High blood pressure, *n* (%)	1 813 (32.5)	5 693 (15.3)
Chronic kidney disease, *n* (%)	324 (5.8)	219 (0.6)
Obesity, *n* (%)	357 (6.4)	1 900 (5.1)
Diabetes, *n* (%)	524 (9.4)	3 062 (8.3)
Treatment received in the year prior to 2^nd^ vaccine dose		
Hydroxychloroquine, *n* (%)	2 466 (44.2)	33 (0.1)
Immunosuppressive drug, *n* (%)	2 802 (50.2)	1 036 (2.8)
Prednisolone	2 123 (38.0)	732 (2.0)
Azathioprine	584 (10.5)	63 (0.2)
Mycophenolic acid	561 (10.0)	49 (0.1)
Methotrexate	462 (8.3)	358 (1.0)
Belimumab	136 (2.4)	0
Cyclophosphamide	8 (0.1)	1 (0.0)
Anti-CD20 mAb	41 (0.7)	0

SLE: systemic lupus erythematosus; q1: first quartile; q3: third quartile; Anti-CD20 mAb: monoclonal antibodies targeting the CD20 (rituximab, ofatumumab, obinutuzumab).

**Figure 3.**
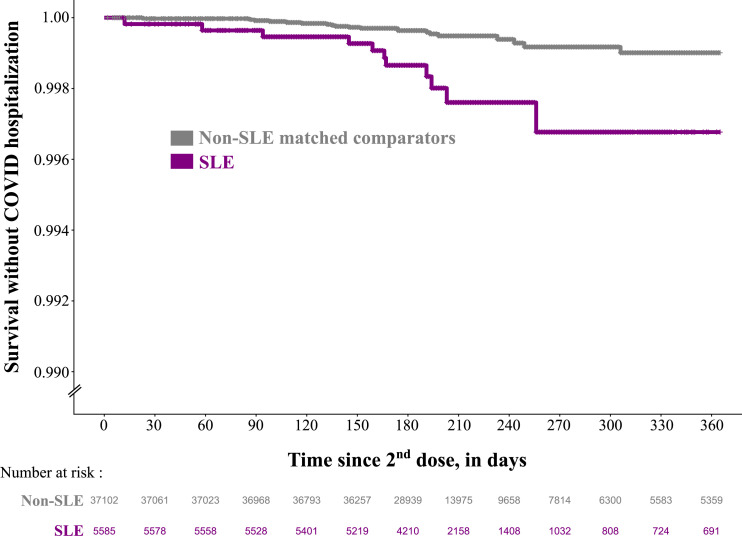
Survival without COVID-19 as a main diagnosis in inpatient care among SLE patients and non-SLE matched comparators from general population who received two doses of mRNA vaccines in Sweden before January 1^st^, 2022, and before any COVID-19 diagnosis in in-or outpatient care (population 2). SLE: systemic lupus erythematosus.

For the main outcome (first hospitalization with COVID-19 as main diagnosis), the median duration of follow-up was 196 days in both groups. The SLE group had 3339 patients-years of follow-up versus 22,745 in the comparator group. Most of the patients were censored because of a third dose (86.0% in the SLE group and 85.0 % in the control group). The number of patients censored because of death or emigration during follow-up was very low in both groups (1.4% in the SLE group and 0.6% in the control group). We observed 11 COVID-19 hopsitalizations (0.20%) in the SLE group versus 20 COVID-19 hospitalizations (0.05%) in the non-SLE comparator (Table 2). The Kaplan-Meier curve representing the survival without COVID-19 hospitalization is displayed in Figure 3. The crude HR of hospitalization with COVID-19 as main diagnosis associated with SLE during the follow-up was 3.76 [95%CI 1.80-7.85]. After adjusting for age, sex, household composition and administrative health region, the HR remained almost unchanged (HR 3.47 [95%CI 1.63-7.39]). We observed more secondary outcomes (any first COVID-19 diagnoses in inpatient or outpatient care during follow-up): 29 (0.5%) in the SLE group and 57 (0.2%) in the non-SLE comparator group (ESM, Figure S3). However, the unadjusted and adjusted HRs associated with SLE were very close to those observed for the primary outcome: 3.52 [95%CI 2.26-5.49] and 3.59 [95%CI 2.30-5.59], respectively.

**Table 2.** SLE patients and non-SLE matched comparators from general population received two doses of mRNA vaccines in Sweden before January 1^st^, 2022, and before any COVID-19 diagnosis in inpatient or outpatient care (population 2).

	SLE *n* = 5585	Non-SLE comparators *n* = 37,102
First hospitalization with COVID as main diagnosis		
Events	11	20
Patients-years	3 339	22 745
Incidence (per 1000 PY) [95%CI]	3.29 [1.82-6.00]	0.88 [0.57-1.36]
Unadjusted HR	3.76 [1.80-7.85]	Ref
Adjusted^a^ HR	3.47 [1.63-7.39]	Ref
Any first COVID-19 diagnoses in inpatient our outpatient care		
Events	29	57
Patients-years	3 333	22 734
Incidence (per 1000 PY) [95%CI]	8.70 [6.05-12.5]	2.51 [1.93-3.25]
Unadjusted HR	3.52 [2.26-5.98]	Ref
Adjusted^a^ HR	3.59 [2.30-5.59]	Ref

HR: hazard ratio; SLE = systemic lupus erythematosus; PY: patients-years; ref: reference; [95% CI]: 95% confidence interval; .

^a^HRs are calculated using a marginal Cox model adjusting for age, sex, household composition and administrative health region.

Of note, we observed only seven COVID-19 related deaths (COVID-19 reported as a cause of death in the Cause of Death Register), six in the comparator group and one in the SLE group. Among these seven deaths, four occurred during a hospital stay with COVID-19 as the main diagnosis.

### Vaccine effectiveness according to the immunosuppressive treatment

**Figure 4.**
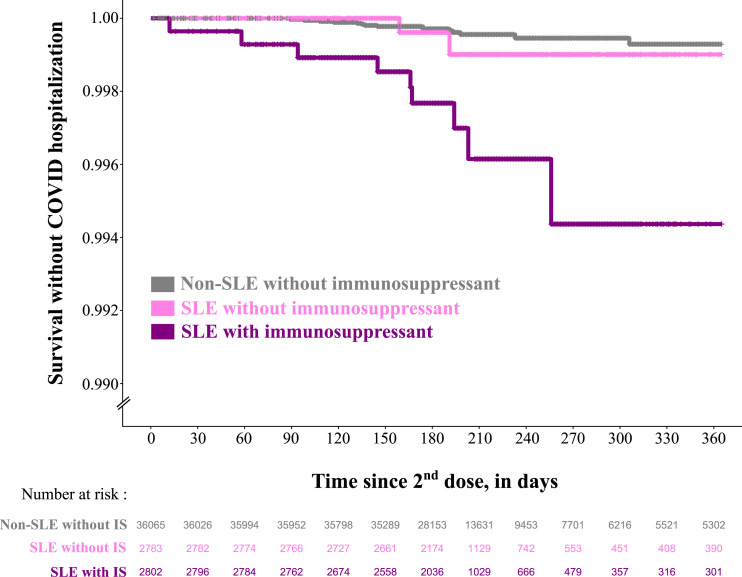
Survival without COVID-19 as a main diagnosis in inpatient care according to the use of immunosuppressant in the year before second vaccine dose among SLE patients and non-SLE matched comparators from the general population who received two doses of mRNA vaccines in Sweden before January 1^st^, 2022, and before any COVID-19 diagnosis in in-or outpatient care. SLE: systemic lupus erythematosus; w/o: without; IS: immunosuppressant.

Among population 2, there were 2802 SLE patients treated with immunosuppressants during the year before their second vaccine dose, 2783 SLE patients not treated with immunosuppressants and 36,065 comparators individuals with no immunosuppressant treatment. We observed that nine out of 11 (81.8%) COVID-19 hospitalizations in the SLE group occurred among those treated with immunosuppressants. Outcome-free survival was very similar between the non-SLE comparator and SLE patients without immunosuppression (Figure 4). This was supported by the results of the Cox model which estimated an unadjusted HR of 1.73 [95%CI 0.40-7.57] for SLE patients without IS and of 8.22 [95%CI 3.60-18.8] for SLE patients treated with IS, compared to non-SLE comparators without IS. After adjusting for age, sex, household composition, health administrative region and number of inpatient or outpatient contacts in 2020, the HRs were qualitatively similar to the unadjusted (1.50 [95%CI 0.34-6.60] for SLE patients without IS and 7.03 [95%CI 3.00-16.46] for SLE patients treated with IS compared to non-SLE comparators). The number of events were too low for us to look for differences according to specific IS treatment (ESM Table S4). Of note, none of the patients who got cyclophosphamide or rituximab were hospitalized with COVID.

### Sensitivity analyses

When using IPCW to account for the censoring mechanism, we did not observe any major differences in the results: the crude HR was 4.47 [95%CI 1.59-12.57] and the adjusted HR was 4.40 [95%CI 1.55-12.5]. When patients were not censored at third dose, we observed 38 (0.7%) in the SLE group and 44 COVID-19 hospitalizations (0.1%) in the comparator group. And the adjusted HR associated with SLE was 5.32 [95%CI 3.42-8.27].

Investigating the effect of the winter 2021-2022 COVID-19 wave in Sweden by adding a time dependent covariate in the model, but we did not find a statistically significant interaction between SLE and the COVID-19 wave period (*p* = 0.18).

## Discussion

We leveraged Swedish population-based, nationwide registers to describe coverage and assess the effectiveness of anti-SARS-CoV-2 vaccination in SLE patients. We found that the uptake of the vaccine was very similar between individuals with and without SLE. The incidence of COVID-19 related hospitalization after vaccination was low in both SLE and comparators, suggesting an overall good effectiveness of the vaccination. However, mRNA vaccines were still less effective in the SLE patients, especially after 6 months of follow-up. The increased risk was observed mainly in the group with a history of immunosuppressive treatment, while SLE patients without immunosuppressive treatment had a risk of COVID-19 hospitalization very similar to that of the general population.

Although this is the first study to assess the anti-SARS-CoV-2 mRNA vaccine’s clinical effectiveness in a real-life setting in SLE patients, our findings are in line with the pre-existing literature about vaccine (anti-SARS-CoV-2 or others) immunogenicity in SLE patients.^6,15,16^ In a previous prospective cohort study,^6^ we observed that after BNT162b2 vaccination, SLE patients had a dramatically decreased humoral response against SARS-CoV-2 variants of concern such as omicron, which was involved in the winter 2021-2022 pandemic wave. However, we found in the current study that patients without immunosuppression had similar COVID-19 hospitalization risk to general population comparators even though the vaccine-induced humoral response is decreased in SLE. This finding could suggest an important role of the cellular, T-cell driven protection induced by the vaccine that was found to be still effective in patients with autoimmune diseases, despite a low humoral response.^7,17^

We observed a very similar pattern in the vaccine uptake between SLE patients and the general population. Of note, in Sweden, the vaccination was scheduled by the central administration and not by the physicians in charge of the patient. Physicians were only allowed to prioritize patients with severe risk factors such as heavy immunosuppression or severe lung disease, that is a small minority of SLE patients. At the beginning of fall 2021, vaccination coverage reached a peak. Although this was true for our study population and similar to what was reported by the Swedish health authorities for the Swedish population,^18^ 9% of SLE patients remaining unvaccinated is an issue given the risk that these patients face regarding COVID-19. Our study was not designed to study the barriers to vaccination in SLE but physicians should be aware that the proportion of unvaccinated people was notably elevated among foreign-born residents and young patients who have already been shown to be more prone to vaccine hesitancy, either with or without autoimmune disease.^19,20^

The maximum difference in vaccine effectiveness between SLE patients and comparators was observed after 6 months of follow-up, which was when the third vaccine dose was recommended, after the protection provided by the first two doses began to decrease. It is likely that this decrease was more marked among immunocompromised individuals such as SLE patients. To account for bias associated with the censoring mechanism, we performed sensitivity analyses using IPCW and removed the third dose from the reason of censoring, which did not change the conclusions of our analysis. Because the 6 months period after the second dose coincided with a COVID-19 wave in Sweden, we looked for an interaction between this wave and SLE, which would have indicated that the wave affected SLE patients and general comparators differently. This interaction was not significant but interaction tests are known to lack power.^21^

Our study was limited by the small number of events observed during the follow-up. Even though this is good news for patients and physicians, it impaired our ability to perform subgroup analysis and to investigate specific immunosuppressive treatments. This work also has the usual limitations of administrative health register studies that rely on ICD-10 codes. Some comorbidities like obesity and diabetes could be under-reported. However, misclassification of these covariates should have only a limited impact on our estimates. We had no information on the SARS-CoV-2 variant involved in the COVID-19 episode. More importantly, we captured diagnoses for COVID-19 only from hospital care, which misses less severe infection. Nevertheless, we believe that it is a strength to examine the more severe outcome, which the vaccine was developed to prevent. We assumed that the only reason that drove the decision to admit was the immediate severity of the patient, but patients with SLE might be more likely to be hospitalized with COVID-19, or to test and seek medical care if they are infected than people without SLE. This would lead to a differential misclassification bias of the outcome and overestimation of the HRs. However, our secondary outcome included diagnoses from outpatient care and results were similar to the primary outcome. The dispensation of immunosuppressive treatment in the year before vaccination might not reflect the state of immunosuppression at time of vaccination and during follow-up. Additionally, some patients on immunosuppressive medications could have been told to pause their treatment around vaccination and for some time after. However, we observed that almost all outcomes in the SLE group occurred in the group using immunosuppressives in the year before the second dose, meaning that our definition was a good proxy for immunosuppression status at vaccination. We cannot exclude the possibility that people on immunosuppressive drugs might have had a lower threshold for hospitalization which would cause differential outcome misclassification, leading to an overestimation of the true effect. In order to have a homogenous population, we excluded patients with a previous COVID-19 diagnosis in the vaccine effectiveness assessment analysis, it is possible that our results may not apply to the whole SLE population and that patients most prone to COVID-19 had already been infected at that time.

In conclusion, vaccination uptake and coverage were similar in Sweden between individuals with SLE and general population comparators of the same age and sex, but that it could still be improved. Even though we observed a very low number of post-vaccination COVID-19 hospitalizations, vaccine effectiveness was diminished in SLE patients, especially among those who recently used or were using immunosuppressive medication. The vaccination scheme could possibly be tailored among patients treated with immunosuppressants. The use of other prophylactic treatment such as monoclonal antibodies should be proposed to immunosuppressant-treated SLE patients in case of reactivation of the pandemic or if mRNA vaccines are developed for other indications.

## Supplemental Material

Supplemental Material - Anti-SARS-CoV-2 mRNA vaccination among patients living with SLE in Sweden: Coverage and clinical effectiveness Supplemental Material for Anti-SARS-CoV-2 mRNA vaccination among patients living with SLE in Sweden: Coverage and clinical effectiveness by Arthur Mageau, Julia F Simard, Elisabet Svenungsson and Elizabeth V Arkema in Lupus
