# Supplementary material for: Anti-SARS-CoV-2 mRNA vaccination among patients living with SLE in Sweden: Coverage and clinical effectiveness
Source: Lupus. 2024 Aug 12;33(11):1192–202. doi: 10.1177/09612033241273052 (PMC11416733; doi:10.1177/09612033241273052)
Supplement: Supplemental Material - Anti-SARS-CoV-2 mRNA vaccination among patients living with SLE in Sweden: Coverage and clinical effectiveness [file sj-pdf-1-lup-10.1177_09612033241273052.pdf]

Anti-SARS-CoV-2 mRNA vaccination among patients living with SLE in Sweden: coverage and clinical effectiveness.

ELECTRONIC SUPPLEMENTARY MATERIALS

Table S1. Definitions of variables used in analyses including International Classification of Diseases (ICD) Swedish revision codes and Anatomical Therapeutic Chemical (ATC) classification system codes.

Variable	Definition	
Exposure		**ICD-10 codes**
Systemic lupus erythematosus	≥2 inpatient or outpatient visits in the NPR with ≥1 visit in a rheumatology, internal medicine, nephrology, dermatology, or pediatric clinic	M32.1–M32.9
**Outcome**		**ICD-10 codes**
COVID-19	- main outcome: hospitalization as main diagnosis in inpatient register<br>- secondary outcome: any diagnoses in inpatient our outpatient care	U07.1, U07.2
**Covariates (diagnoses)**		**ICD-10 codes**
Hypertension	≥1 visit in the NPR before start of follow-up	I10.X
Chronic kidney disease	≥1 visit in the NPR before start of follow-up	N18.3, N18.4, N18.5
Obesity	≥1 visit in the NPR before start of follow-up	E66.X
Diabetes mellitus	≥1 visit in the NPR or ≥1 dispensation of an antidiabetic drug (see below) before start of follow-up	E10.X, E11.X, E12.X, E13.X, E14.X, O24.X
**Drugs**		**ATC codes**
Hydroxychloroquine	≥2 dispensations in the year before first dose	P01BA02
Prednisolone	≥2 dispensations in the year before first dose	H02AB06
Azathioprine	≥2 dispensations in the year before first dose	L04AX01
Mycophenolic acid	≥2 dispensations in the year before first dose	L04AA06
Methotrexate	≥2 dispensations in the year before first dose	L04AX03
Belimumab	≥1 dispensation in the PDR OR ≥1 infusion (in SRQ or infusion procedure “DT016” + ATC code in the NPR) during the last 6 months before first dose	L04AA26
Cyclophosphamide	≥1 dispensation in the PDR OR ≥1 infusion (in SRQ or infusion procedure “DT016” + ATC code in the NPR) during the last 6 months before first dose	L01AA01
Anti-CD20 monoclonal antibody	≥1 dispensation in the PDR OR ≥1 infusion (in SRQ or infusion procedure “DT016” + ATC code in the NPR) during the last 6 months before first dose	L01FA01, L04AA52, L01FA03
Antidiabetic drug	≥1 dispensation in the PDR before first dose	A10.X
**Other variables**		
Household composition	- If one child < 18 years old on January 1, 2021, in the MR → living with (a) child(ren)<br>- If no child < 18 years but married or living with a partner in 2020 in the TPR → "Living with a partner, without having any child < 18 years old "<br>- else: "living alone"	
Number of contacts in inpatient or non-primary outpatient care in 2020	Number of visits in the NPR in 2020	

TPR; Total patient register; NPR: national patient register; SRQ: Swedish rheumatology quality register; PDR: prescribed drug register; MR: multigeneration register

Table S2: Characteristics of SLE patients on Jan 1, 2021, who received or did not receive any anti-SARS-CoV-2 vaccine dose by December 31, 2021, in Sweden.

	**SLE NOT VACCINATED n= 650**	**SLE VACCINATED n= 6 703**
**Demographics**		
Age at January 1st 2021, years, median [q1-q3]	47.4 [35.6-58.5]	57.9 [45.0-71.0]
Female, n (%)	576 (88.6 %)	5 801 (86.5%)
Born out of Sweden, n (%)	226 (34.8)	1 096 (16.4)
Education level, n (%) :		
0-9 years	131 (20.2)	1 184 (17.7)
10-12 years	302 (46.5)	2 850 (42.5)
≥ 10-13 years	202 (31.1)	2 626 (39.2)
missing	15 (2.3)	43 (0.6)
Number of children, median [q1-q3]	1 [0-2]	2 [1-2]
Household composition, n (%) :		
Living alone	313 (48.2)	2 895 (43.2)
Living with children	210 (32.3)	1 426 (21.3)
Living with partner and without child	127 (19.5)	2 382 (35.5)
Health administrative region, n (%):		
Stockholm	172 (26.5)	1 392 (20.8)
Uppsala-Örebro	114 (17.5)	1 469 (21.9)
North	46 (7.1)	642 (9.6)
West	129 (19.8)	1 221 (18.2)
South	123 (18.9)	1 114 (16.6)
Southeast	66 (10.2)	865 (12.9)
**SLE-related variables**		
Time since SLE diagnosis, in years, median [q1-q3]	9.9 [4.4-17.9]	12.8 [6.3-18.9]
Hydroxychloroquine*, n (%):	244 (37.5)	2964 (44.2)
Prednisolone*, n (%):	230 (35.4)	2 609 (38.9)
DMARDs*, n (%):	142 (21.9)	1 857 (27.7)
**COVID-19**		
COVID-19 diagnosis in inpatient or outpatient care before Dec 31, 2021, n (%):	39 (6.0)	198 (3.0)
**Comorbidity history**		
High blood pressure, n (%):	162 (24.9)	2 244 (33.5)
Chronic kidney disease, n (%):	27 (4.2)	392 (5.8)
Diabetes, n (%):	50 (7.7)	593 (8.8)
Obesity, n (%):	49 (7.5)	433 (6.5)

SLE: systemic lupus erythematosus; q1: first quartile; q3: third quartile; DMARDs: disease modifying anti-rheumatic drug (azathioprine, methotrexate, or mycophenolic acid). * 2 or more dispensations in the year prior to start of follow-up (Jan 1, 2021)

Figure S3: Survival without COVID-19 as any diagnosis in in- or outpatient care (secondary outcome) among SLE patients and matched general population comparators who received two doses of mRNA vaccines in Sweden before January 1st, 2022, and before any COVID-19 diagnosis in in- or outpatient care (population 2).

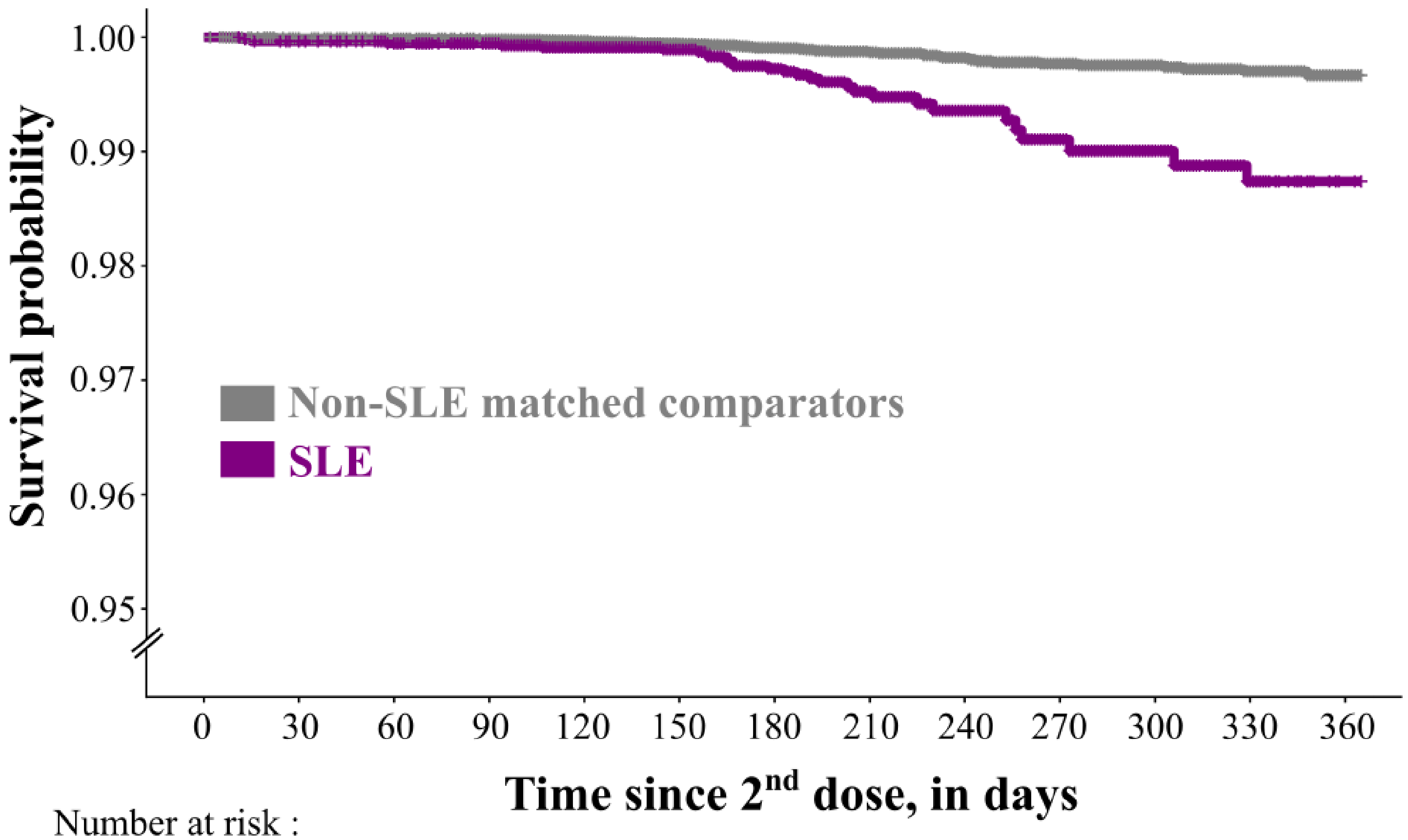

Number at risk :

Non-SLE	37102	37059	37020	36964	36788	36247	28921	13961	9640	7797	6285	5567	5342
SLE	5585	5577	5557	5527	5399	5217	4203	2149	1397	1022	798	712	679

Table S4: Comparison of immunosuppressant use between SLE patients who experienced or who did not experience the main outcome (COVID-19 hospitalization) among those who had received an immunosuppressive drug within the first year before first vaccine dose.

	**Not hospitalized for COVID-19 n=2,793**	**Hospitalized for COVID-19 n=9**
Hydroxychloroquine, n (%):	1 497 (53.6)	3 (33.3)
Immunosuppressive drug, n (%):		
Prednisolone	2 115 (75.7)	8 (88.9)
Azathioprine	582 (20.8)	2 (22.2)
Mycophenolic acid	559 (20.0)	2 (22.2)
Methotrexate	460 (16.5)	2 (22.2)
Belimumab	135 (4.8)	1 (11.1)
Cyclophosphamide	8 (0.3)	0
Rituximab	41 (1.5)	0
